# Systematic review and meta-analysis of the etiology of heavy menstrual bleeding in 2,770 adolescent females

**DOI:** 10.1186/s12905-024-02921-7

**Published:** 2024-02-20

**Authors:** Erin M. Hall, Ana E. Ravelo, Stephen C. Aronoff, Michael T. Del Vecchio

**Affiliations:** 1 Department of Pediatrics, University of Washington, Seattle, WA USA; 2 Department of Pediatrics, Brown University, Providence, RI USA; 3 Department of Pediatrics, Lewis Katz School of Medicine at Temple University, Philadelphia, PA USA

**Keywords:** Adolescents, Menorrhagia, Abnormal uterine bleeding, Heavy menstrual bleeding, Systematic review, PALM-COEIN

## Abstract

**Background:**

Adolescent heavy menstrual bleeding(HMB), menorrhagia or abnormal uterine bleeding commonly occur in adolescent women. The differential diagnosis can be challenging. The pneumonic: PALM-COEIN (polyp, adenomyosis, leiomyoma, malignancy and hyperplasia, coagulopathy, ovulatory dysfunction, endometrial, iatrogenic, and not yet classified), is commonly used but it does not stratify as to the likelihood of a disorder. We have sought to develop a probability-based differential diagnosis for Adolescent HMB, menorrhagia or abnormal uterine bleeding.

**Methods:**

A comprehensive literature search was conducted using PubMed, EMBASE, and SCOPUS databases. Case series describing adolescents from 10–19 years of age with HMB, menorrhagia or abnormal uterine bleeding was acceptable if: more than 10 patients were included; editorials, case reports, and secondary sources such as review articles, or book chapters were excluded. No language filter was used, but an English abstract was required. The etiology of HMB, menorrhagia or abnormal uterine bleeding, and the country of origin was extracted from articles that met inclusion criteria. Cumulative rate estimates were determined by Bayesian probability modeling.

**Results:**

Seventeen full text articles were reviewed in detail; 2,770 patients were included. The most frequent causes of HMB were Ovarian Uterine Disorders (23.7%; 95% CredI 22–25.5%), Coagulation Disorders (19.4%; 95% CredI 17.8—21.1%), and Platelet Disorders (6.23%; 95% CredI 5.27–7.27%) with 45.9% (95% CredI 43.8—47.%9) of the cases of indeterminate origin.

**Conclusions:**

The leading causes of HMB in healthy adolescent females were varied. The sub-analysis identified distinct etiologies, suggesting that multiple factors must be considered in the evaluation of HMB. While PALM-COEIN (polyp, adenomyosis, leiomyoma, malignancy and hyperplasia, coagulopathy, ovulatory dysfunction, endometrial, iatrogenic, and not yet classified) provides us with a comprehensive picture of the possible causes of HMB in females, this systematic review assigns probabilities to the etiologies of HMB in adolescent females, providing physicians with a more focused and efficient pathway to diagnosis.

## Background

Among female adolescents, menorrhagia or heavy menstrual bleeding (HMB), has an estimated prevalence of 37% [1]. It is important to note the complexities in terminology when discussing heavy or abnormal menstrual bleeding. Differing terminology has led to difficulty interpreting the clinical literature. FIGO (Federation of Gynecology and Obstetrics) has recommended abandoning the terms menorrhagia, as well as disordered uterine bleeding (DUB), given they often confuse both a diagnosis and a symptom [2]. However, a large portion of the literature that addresses heavy or abnormal menstrual bleeding, actively uses these controversial terms.

Heavy menstrual bleeding (HMB), defined as excessive menstrual blood loss that interferes with a woman’s physical, social, emotional, or material quality of life, is currently classified according to the FIGO abnormal uterine bleeding system 2 PALM-COEIN classification system [2, 3]: (polyp, adenomyosis, leiomyoma, malignancy and hyperplasia, coagulopathy, ovulatory dysfunction, endometrial, iatrogenic, and not otherwise classified). This system, although useful, is not probability based, making it difficult for the physician to develop an efficient diagnostic plan.

Adolescent HMB is typically related to anovulation, however, other causes such as an underlying bleeding disorder must be considered when a healthy female experiences HMB at menarche or during adolescence. While the frequency of bleeding disorders in the general population is estimated to be 1–2%, bleeding disorders are found in approximately 20% of adolescent girls who present for evaluation for HMB and in 33% of adolescent girls hospitalized for HMB [4]. The purpose of this systematic review was to (1) determine the general categories of HMB across a wide spectrum of patients and; (2) determine the relative prevalence of specific etiologies using a probability based methodology.

## Methods

This systematic review followed The Preferred Reporting Items in Systematic Reviews and Meta-analysis (PRISMA) guidelines [5].

### Literature search and inclusion criteria

A comprehensive literature search was conducted using the PubMed, EMBASE, and SCOPUS databases. The search items “Abnormal uterine bleeding” OR “menorrhagia” OR “heavy menstrual bleeding” AND “adolescents” were input. Editorials, case reports, review articles, book chapters, and studies with less than 10 patients were excluded. English abstracts were required. No date filter was used in the PubMed and EMBASE database searches. In the SCOPUS search engine, publication data was filtered to include only articles published from 2020 onward to search for newer publications and abstracts. The literature search was conducted by two authors on two separate occasions (August 2021, September 2021). The bibliographies from each of the databases were uploaded into the RefWorks program and exact duplicates were excluded. Each abstract was reviewed independently by two authors (EMH, AER) to identify those studies that met inclusion criteria (Table 1); conflicts were resolved by a third author (MTD). Throughout the initial search, relevant review articles were identified, and their bibliographies were reviewed also for studies that could qualify for this review. Full-text articles were reviewed using for inclusion and exclusion criteria.

**Table 1:** Inclusion and exclusion criteria

I. Title contains "Menorrhagia", "Abnormal Uterine Bleeding" or "Heavy Menstrual Bleeding"
II. Minimum number of studied children is 10
III, Children are age 0–21 years old
IV. Study entry criteria requires review of all children with menorrhagia without another co-existing diagnosis or treatment modality. For example, studies that examined the etiology of menorrhagia from only children with known bleeding disorders were excluded
V. Not a case report
VI. Not a review article
VII. Not an editorial
VIII. No language filter was utilized, but an English abstract was required

### Data extraction

Following the final selection of articles, the etiologies of HMB, menorrhagia or abnormal uterine bleeding (focusing on abnormal degree of bleeding and not abnormal bleeding intervals), and the number of adolescents affected by each different etiology were recorded for each study.

### Data synthesis

A Bayesian methodology was employed to determine the rate estimates and the associated 95% credible intervals for each disease entity; the beta distribution was used as the conjugate prior to the conditional likelihood distribution for each rate estimate determined from the extracted data [6, 7]. All calculations were performed in the R environment.

## Results

### Study search and selection

**Fig. 1:**
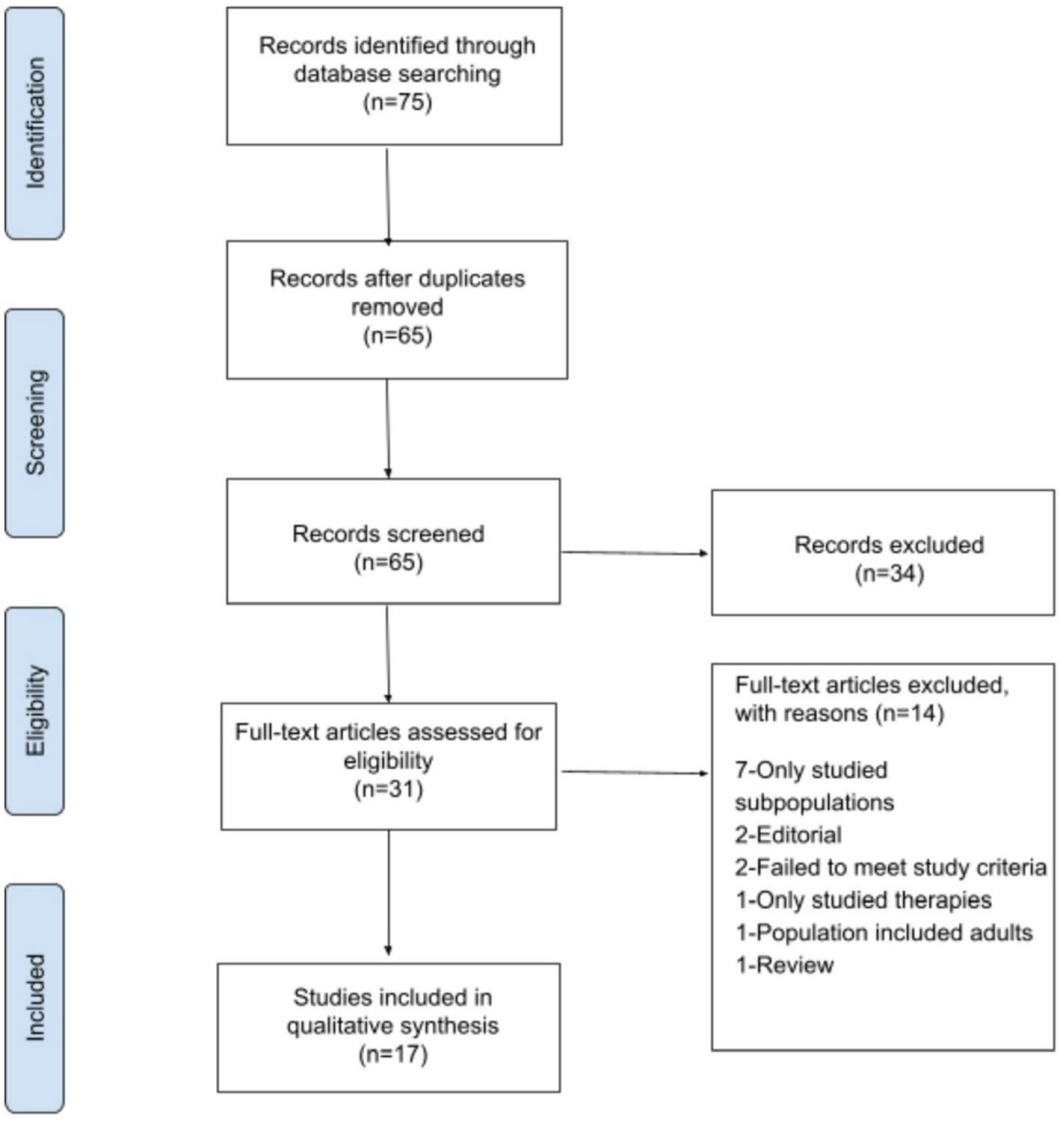
Literature search results. Adapted from Moher et. al. (PLoS Med 2009;6:e1000097) [4].

Figure 1 demonstrates the results of the systematic review. The database searches provided a total of 75 results. After exact duplicates were removed, abstracts of 65 articles were reviewed. After exclusion of abstracts that did not meet inclusion criteria a total of 31 full-text articles were examined in detail. Fourteen full text articles were excluded: 7 only studied subpopulations of patients (ie PCOS only), 2 were editorials, 2 failed to meet study criteria once the methods section was examined, 1 only studied therapies, 1 included adults in the study who could not be separated from those less than 19 years of age, and 1article was a secondary review.

A total of 17 articles (published between 1998–2020) met inclusion criteria and were analyzed for this study.

### Study characteristics

Table 2 summarizes the data extracted from the 17 studies. A total of 2,770 subjects were identified. The largest study included 24% of the patient sample. The categories of entities causing HMB in patients ages 10–19 are shown in Table 3.

**Table 2:** Article extraction

First Author	Year Published	Study Design	Country	No. Of patients	Percentage of Total patients (%)
Aguirre [7]	2020	Prospective	Chile	93	3.4%
Alaqzam [8]	2018	Retrospective	USA	73	2.6%
Haberal [9]	2008	Retrospective	Turkey	120	4.3%
Hutspardol [10]	2010	Prospective	Thailand	28	1.0%
Jain [11]	2020	Prospective	USA	200	7.2%
Khamees [12]	2015	Prospective	USA	673	24.3%
Mikhail [13]	2007	Retrospective	USA	61	2.2%
Mills [14]	2014	Retrospective	USA	114	4.1%
O'Brien [15]	2019	Retrospective	Australia	124	4.5%
Oral [16]	2002	Retrospective	Turkey	25	0.9%
Rathod [17]	2016	Prospective	India	97	3.5%
Rosen [18]	2020	Retrospective	USA	258	9.3%
Seravalli [19]	2013	Retrospective	Italy	113	4.1%
Sharma [20]	2015	Prospective	India	167	6.0%
Smith [21]	1998	Retrospective	USA	179	6.5%
Zia [22]	2020	Prospective	USA	200	7.2%
Zia [23]	2018	Prospective	USA	248	8.9%

**Table 3:** Overview of etiologies

Etiologies	n	MLE	LCredL	UCredL
Indeterminate	1030	45.9	43.8	47.9
Ovarian Uterine Disorders	532	23.7	22	25.5
Coagulation Disorders	436	19.4	17.8	21.1
Platelet Disorders	140	6.23	5.27	7.27
Unspecified Bleeding Disorder	46	2.05	1.5	2.67
Drug Induced Bleeding	16	0.712	0.408	1.1
Bleeding Secondary Renal Disease	11	0.49	0.245	0.818
Infection	10	0.445	0.214	0.76
Isolated Increased Bleeding Time	8	0.356	0.154	0.641
Chemotherapy Recipient	5	0.223	0.0724	0.456
Exon 28 Polymorphism	4	0.178	0.0486	0.39
Fanconi Anemia	3	0.134	0.0276	0.321
Aplastic Anemia	2	0.089	0.0108	0.248
Ehlers Danlos Syndrome	2	0.089	0.0108	0.248
VWD and Platelet Dysfunction	1	0.0445	0.00113	0.164

Almost half of the cases had no identifiable etiology (45.9%, 95%CredI 43.8- 47.9). Ovarian Uterine Disorders (23.7%; 95% CredI 22–25.5), Coagulation Disorders (19.4%; 95% CredI 17.8—21.1), and Platelet Disorders (6.23%; 95% CredI 5.27–7.27). 45.9% (95% CredI 43.8—47.9) accounted for most cases with an identified etiology.

Among the ovarian uterine disorders identified, anovulatory bleeding (98.7%, 95% CredI 97.6—99.5) accounted for almost all of the cases (Table 4); endometriosis (0.564%; 95% CredI 0.117—1.35) and polycystic ovary syndrome (0.564%; 95% CredI 0.117 – 1.35) were encountered rarely. Among coagulopathies, von Willebrand’s Disease (88.1, 95% CredI 84.9 – 90.9) accounted for the vast majority of cases; Factor 8 Deficiency (6.19%; 95% CredI 4.13 – 8.64) and Clotting Factor Deficiencies (4.13%; 95% CredI 2.47 – 6.18) occurred less commonly (Table 5). As a group, platelet abnormalities accounted for a small percentage of patients with HMB; Platelet Function Disorders (37.9%; 95% CredI 30 – 46), Inherited Thrombocytopenia (17.1%; 95% CredI 11.4 – 23.8) and Platelet Qualitative Disorders (13.6%; 95% CredI 8.43 – 19.7) were the most common platelet disorders identified (Table 6).

**Table 4:** Etiologies of uterine disorders

Uterine Disorders	n	MLE	LCredL	UCredL
Anovulatory Bleeding	525	98.7	97.6	99.5
Endometriosis	3	0.564	0.117	1.35
PCOS	3	0.564	0.117	1.35
Uterine Polyps	1	0.188	0.00477	0.692

**Table 5:** Etiologies of Coagulopathies

Coagulopathies	n	MLE	LCredL	UCredL
Von Willebrand Disease	384	88.1	84.9	90.9
Factor 8 Deficiency	27	6.19	4.13	8.64
Clotting Factor Deficiency	18	4.13	2.47	6.18
Other Coagulation Defects	3	0.688	0.142	1.65
Syptomatic Hemophilia Carrier	2	0.459	0.0557	1.27
Factor 11 Deficiency	1	0.229	0.00582	0.844
Factor 9 Deficiency	1	0.229	0.00582	0.844

**Table 6:** Etiologies of platelet abnormalities

Platelet Abnormalities	n	MLE	LCredL	UCredL
Platelet Function Disorder	53	37.9	30	46
Inherited Thrombocytopenia	24	17.1	11.4	23.8
Platelet Qualitative Disorder	19	13.6	8.43	19.7
ITP	12	8.57	4.54	13.7
Platelet Quantitative Disorder	7	5	2.05	9.16
Dysfunctional Platelet Aggregation	6	4.29	1.6	8.19
Multiple Platelet Secretion Defects	4	2.86	0.79	6.18
Unspecified Thrombocytopenia	4	2.86	0.79	6.18
Other Immune Thrombocytopenia	2	1.43	0.175	3.94
Multiple Platelet Aggregation Defects	1	0.714	0.0182	2.62
Platelet Secretion Defects	1	0.714	0.0182	2.62
Aggregation Secretion Defects	7	5	2.05	9.16

## Discussion

A systematic review of HMB in patients ages 10–19 yielded 17 full text articles describing 2,770 adolescent females. These patients spanned the age range of 10–19 years old. Although PALM-COEIN [3] is a useful tool to organize the etiologies of HMB, it fails to provide a priori probabilities for each entity or to estimate the number of patients with no identifiable etiology. The present study identifies the etiologies of HMB in a combined cohort of more than 2000 patients, assigns relative probabilities to the etiologies of HMB in adolescents and that over 40% of individuals will have no identifiable etiology. Among patients with an identifiable etiology for HMB, Ovarian Uterine disorders (23.7%; 95% CredI 22–25.5), Coagulation Disorders (19.4%; 95% CredI 17.8—21.1), and Platelet Disorders (6.23%; 95% CredI 5.27–7.27) were the most common systematic disorders. Within these categories, anovulatory bleeding, von Willebrand’s Disease and platelet function disorders were most common. Per Graham et al., the comprehensive workup of menorrhagia should include ruling out a bleeding disorder with the help of a multidisciplinary team that includes hematology, gynecology, an adolescent medicine specialist, and a primary care provider [8].

As a systematic review, this article has inherent limitations. The etiologies of HMB listed in the selected articles were taken as they were reported in their respective studies. This resulted in vague etiologies, such as unspecified thrombocytopenia and aggregation secretion defects. Additionally, the availability of diagnostic resources, criteria, and thoroughness of evaluation were not standardized across institutions, resulting in the large number of indeterminate etiologies of HMB. This also raises the issue of diagnostic consistency. While the FIGO classification systems for nomenclature, symptoms, and classification of potential causes of abnormal uterine bleeding are excellent resources, this list of possible etiologies is a reasonable starting point for practitioners when a risk-based approach is desired [9]. Future studies could expand upon our data and break up probable etiologies by regions (urban versus rural) and type of hospital where the patient is presenting to (tertiary referral versus frontline institution), given that management would likely differ. Lastly, like the two FIGO classification systems, our probability based differential diagnosis for abnormal uterine bleeding should be subject to ongoing and regular review, to ensure incorporation of new research and analysis [9].

## Conclusions

The present study, a systematic review found the causes of HMB in healthy adolescent females were varied. The sub-analysis identified distinct etiologies, suggesting that multiple factors must be considered in the evaluation of HMB. While PALM-COEIN (polyp, adenomyosis, leiomyoma malignancy and hyperplasia, coagulopathy, ovulatory dysfunction, endometrial, iatrogenic, not yet classified) provides us with a comprehensive picture of the possible causes of HMB in females, this systematic review assigns probabilities to the etiologies of HMB in adolescent females, providing physicians with a more focused and efficient pathway to diagnosis.

## Data Availability

The datasets used and/or analyzed during the current study available from the corresponding author on reasonable request.

## Abbreviations

HMB: Heavy Menstrual Bleeding
PALM-COEIN: Polyp, adenomyosis, leiomyoma, malignancy and hyperplasia, coagulopathy, ovulatory dysfunction, endometrial, iatrogenic, and not yet classified
PRISMA: Preferred Reporting Items for Systematic reviews and Meta-Analysis
CredI: Credible Intervals
